# Supplementary material for: Health insurance and financial hardship in cancer survivors during the COVID-19 pandemic
Source: PLoS One. 2022 Aug 5;17(8):e0272740. doi: 10.1371/journal.pone.0272740 (PMC9355233; doi:10.1371/journal.pone.0272740)
Supplement: S1 Table — (DOCX) [file pone.0272740.s001.docx]

**S1 Table. Non-respondent sociodemographic characteristics (N=11,749).**

|  | Total  N=11,749 |
| --- | --- |
| Age |  |
| 19-35 | 1057 (9.0) |
| 36-55 | 4024 (34.3) |
| 56-75 | 5682 (48.4) |
| ≥ 76 | 876 (7.5) |
| Unknown | 110 (0.9) |
| Race and ethnicity |  |
| White | 5866 (49.9) |
| Black/African American | 3453 (29.4) |
| Hispanic/Latino | 954 (8.1) |
| Other | 827 (7.0) |
| Unknown | 649 (5.5) |
| Household income |  |
| ≤$47,999 | 9062 (77.1) |
| >$47,999 | 2352 (20.0) |
| Unknown | 335 (2.9) |
