# Supplementary material for: Health insurance and financial hardship in cancer survivors during the COVID-19 pandemic
Source: PLoS One. 2022 Aug 5;17(8):e0272740. doi: 10.1371/journal.pone.0272740 (PMC9355233; doi:10.1371/journal.pone.0272740)
Supplement: S2 Table — (DOCX) [file pone.0272740.s002.docx]

**S2 Table. Adjusted model results estimating relative risk of trouble paying for non-healthcare necessities in under-resourced cancer survivors (N=1,437).**

|  | Food | Household supplies | Housing (rent/mortgage) | Utilities | Phone | Internet/data | Car, gas, transportation | Childcare, elder care |
| --- | --- | --- | --- | --- | --- | --- | --- | --- |
|  | RR (95% CI) | RR (95% CI) | RR (95% CI) | RR (95% CI) | RR (95% CI) | RR (95% CI) | RR (95% CI) | RR (95% CI) |
| Health insurance status |  |  |  |  |  |  |  |  |
| Medicaid | Ref. | Ref. | Ref. | Ref. | Ref. | Ref. | Ref. | Ref. |
| ACA Marketplace | 0.82 (0.63-1.06) | 0.85 (0.64-1.14) | 0.97 (0.74-1.27) | 0.79 (0.60-1.04) | 0.88 (0.62-1.25) | 0.72 (0.47-1.12) | 0.84 (0.62-1.14) | 0.61 (0.21-1.79) |
| Employer-sponsored | 0.74 (0.61-0.90) | 0.67 (0.53-0.85) | 0.69 (0.55-0.86) | 0.61 (0.49-0.76) | 0.60 (0.45-0.81) | 0.60 (0.43-0.84) | 0.68 (0.54-0.87) | 0.62 (0.32-1.22) |
| Medicare | 0.71 (0.60-0.85) | 0.68 (0.55-0.84) | 0.72 (0.58-0.90) | 0.63 (0.52-0.77) | 0.56 (0.43-0.73) | 0.68 (0.51-0.91) | 0.57 (0.45-0.72) | 0.57 (0.27-1.23) |
| Uninsured | 0.86 (0.64-1.16) | 0.88 (0.64-1.22) | 0.97 (0.71-1.31) | 0.82 (0.59-1.13) | 1.09 (0.79-1.51) | 0.93 (0.60-1.44) | 0.98 (0.72-1.34) | 0.42 (0.10-1.44) |
| Other / unknown | 0.78 (0.64-1.16) | 0.76 (0.56-1.03) | 0.75 (0.56-1.02) | 0.83 (0.65-1.06) | 0.89 (0.64-1.23) | 0.86 (0.58-1.30) | 0.76 (0.55-1.34) | 0.51 (0.18-1.65) |
| Age |  |  |  |  |  |  |  |  |
| 19-35 | Ref. | Ref. | Ref. | Ref. | Ref. | Ref. | Ref. | Ref. |
| 36-55 | 0.98 (0.75-1.28) | 1.13 (0.79-1.62) | 1.09 (0.78-1.52) | 1.13 (0.84-1.52) | 0.88 (0.62-1.25) | 0.86 (0.57-1.28) | 0.83 (0.63-1.11) | 0.32 (0.17-0.61) |
| 56-75 | 0.81 (0.61-1.07) | 0.96 (0.67-1.38) | 0.81 (0.57-1.16) | 0.83 (0.61-1.13) | 0.73 (0.51-1.05) | 0.69 (0.45-1.04) | 0.65 (0.48-0.89) | 0.10 (0.05-0.23) |
| ≥ 76 | 0.41 (0.23-0.76) | 0.71 (0.38-1.31) | 0.60 (0.31-1.17) | 0.47 (0.24-0.90) | 0.43 (0.19-0.96) | 0.20 (0.06-0.66) | 0.51 (0.26-0.99) | 0.21 (0.05-0.83) |
| Sex |  |  |  |  |  |  |  |  |
| Male | Ref. | Ref. | Ref. | Ref. | Ref. | Ref. | Ref. | Ref. |
| Female | 0.90 (0.77-1.06) | 1.19 (0.96-1.47) | 1.04 (0.83-1.30) | 0.95 (0.80-1.13) | 1.04 (0.82-1.31) | 1.06 (0.82-1.38) | 0.89 (0.72-1.11) | 1.27 (0.64-2.53) |
| Race and ethnicity |  |  |  |  |  |  |  |  |
| White | Ref. | Ref. | Ref. | Ref. | Ref. | Ref. | Ref. | Ref. |
| Black/African American | 1.45 (1.26-1.66) | 1.46 (1.24-1.72) | 1.31 (1.11-1.54) | 1.53 (1.31-1.78) | 1.43 (1.16-1.77) | 1.27 (1.00-1.61) | 1.30 (1.08-1.57) | 2.00 (1.11-3.63) |
| Hispanic/Latino | 1.13 (0.91-1.41) | 1.19 (0.92-1.55) | 1.03 (0.80-1.33) | 1.24 (0.98-1.57) | 1.06 (0.78-1.45) | 1.09 (0.77-1.55) | 1.28 (0.98-1.66) | 1.00 (0.39-2.56) |
| Other | 1.12 (0.85-1.47) | 1.31 (0.98-1.76) | 1.12 (0.83-1.51) | 1.14 (0.85-1.54) | 1.25 (0.89-1.76) | 1.44 (1.01-2.06) | 1.39 (1.04-1.86) | 2.99 (1.43-6.26) |
| Education |  |  |  |  |  |  |  |  |
| < High school | Ref. | Ref. | Ref. | -- | -- | -- | Ref. | -- |
| High school degree | 0.90 (0.66-1.22) | 0.98 (0.68-1.41) | 1.12 (0.71-1.77) | -- | -- | -- | 1.12 (0.72-1.74) | -- |
| Some college | 0.89 (0.65-1.21) | 1.03 (0.72-1.48) | 1.30 (0.83-2.03) | -- | -- | -- | 1.17 (0.76-1.82) | -- |
| ≥ Bachelor’s degree | 0.82 (0.60-1.12) | 0.85 (0.59-1.23) | 1.05 (0.66-1.66) | -- | -- | -- | 0.93 (0.59-1.46) | -- |
| Employment status |  |  |  |  |  |  |  |  |
| Employed | Ref. | Ref. | Ref. | Ref. | Ref. | Ref. | Ref. | -- |
| Retired | 0.55 (0.42-0.73) | 0.48 (0.34-0.68) | 0.34 (0.23-0.49) | 0.38 (0.27-0.54) | 0.35 (0.22-0.56) | 0.40 (0.25-0.65) | 0.55 (0.37-0.80) | -- |
| Disabled | 1.13 (0.94-1.35) | 1.28 (1.03-1.60) | 0.95 (0.77-1.16) | 0.96 (0.79-1.16) | 0.98 (0.75-1.27) | 1.00 (0.74-1.34) | 1.36 (1.07-1.73) | -- |
| Unemployed/other | 0.99 (0.81-1.22) | 1.08 (0.84-1.38) | 1.17 (0.95-1.44) | 0.97 (0.78-1.20) | 1.24 (0.95-1.62) | 1.16 (0.84-1.59) | 1.24 (0.95-1.61) | -- |
| Household income |  |  |  |  |  |  |  |  |
| ≤ $47,999 | Ref. | Ref. | Ref. | Ref. | Ref. | Ref. | Ref. | -- |
| > $47,999 | 0.94 (0.81-1.10) | 0.81 (0.66-0.99) | 0.82 (0.67-1.00) | 0.69 (0.56-0.84) | 0.62 (0.47-0.81) | 0.64 (0.47-0.86) | 0.72 (0.58-0.90) | -- |
| RUCA |  |  |  |  |  |  |  |  |
| Urban | Ref. | Ref. | Ref. | Ref. | Ref. | Ref. | Ref. | -- |
| Rural | 1.07 (0.89-1.30) | 1.05 (0.83-1.33) | 0.63 (0.46-0.87) | 0.80 (0.62-1.03) | 0.75 (0.52-1.10) | 0.85 (0.57-1.25) | 1.14 (0.88-1.46) | -- |
| Cancer type |  |  |  |  |  |  |  |  |
| Breast | Ref. | Ref. | Ref. | -- | -- | -- | Ref. | -- |
| Genitourinary | 0.74 (0.49-1.12) | 1.12 (0.71-1.75) | 1.01 (0.65-1.56) | -- | -- | -- | 0.56 (0.30-1.07) | -- |
| Gynecological | 0.89 (0.59-1.33) | 0.78 (0.46-1.33) | 0.60 (0.33-1.08) | -- | -- | -- | 1.17 (0.74-1.85) | -- |
| Gastrointestinal | 0.97 (0.73-1.29) | 1.14 (0.85-1.55) | 0.91 (0.66-1.26) | -- | -- | -- | 1.17 (0.85-1.60) | -- |
| Hematologic | 1.08 (0.92-1.28) | 1.11 (0.91-1.34) | 0.84 (0.68-1.03) | -- | -- | -- | 0.97 (0.78-1.20) | -- |
| Other | 1.09 (0.93-1.29) | 0.99 (0.81-1.21) | 0.88 (0.72-1.07) | -- | -- | -- | 0.94 (0.76-1.17) | -- |
| Comorbidity count* |  |  |  |  |  |  |  |  |
| 0 | Ref. | Ref. | Ref. | Ref. | Ref. | Ref. | Ref. | Ref. |
| 1-2 | 1.09 (0.93-1.27) | 1.09 (0.91-1.32) | 0.99 (0.83-1.18) | 1.01 (0.85-1.20) | 0.89 (0.70-1.12) | 0.89 (0.68-1.15) | 0.92 (0.75-1.13) | 0.87 (0.46-1.65) |
| ≥ 3 | 1.39 (1.20-1.61) | 1.62 (1.36-1.93) | 1.28 (1.07-1.53) | 1.39 (1.18-1.64) | 1.45 (1.18-1.79) | 1.49 (1.18-1.89) | 1.45 (1.20-1.74) | 1.51 (0.84-2.71) |

*Count of comorbidities other than cancer

RR=relative risk, CI=confidence interval, ACA=Affordable Care Act, RUCA=Rural-Urban Commuting Area
